# The efficacy of intraarticular viscosupplementation after arthroscopic partial meniscectomy: a randomized controlled trial

**DOI:** 10.1186/s12891-021-04990-3

**Published:** 2022-01-04

**Authors:** Kyoung Ho Yoon, Woo Seung Wan, Yoon-Seok Kim, Jae-Young Park

**Affiliations:** 1 Department of Orthopaedic Surgery, Kyung Hee University Hospital, Seoul, Republic of Korea; 2 Department of Orthopaedic Surgery, Uijeongbu Eulji Medical Center, Eulji University School of Medicine, 712, Dongil-ro, Uijeongbu-si, Gyeonggi-do Republic of Korea

**Keywords:** Viscosupplementation, Hyaluronic acid, Arthroscopy, Meniscectomy

## Abstract

**Background:**

This study aimed to evaluate the efficacy of viscosupplementation after arthroscopic partial meniscectomy.

**Method:**

A randomized controlled trial of 47 patients who underwent arthroscopic partial meniscectomy was conducted between March 2020 and March 2021. Patients were randomized into two groups: a viscosupplementation group (*n* = 23) and a control group (*n* = 24). A single-dose intraarticular hyaluronic acid injection was used as viscosupplementation. The 100 mm visual analogue scale (VAS) for pain assessment was measured at baseline and at 1 day, 2 weeks, 6 weeks, and 3 months post-surgery. The International Knee Documentation Committee (IKDC), Tegner, Lysholm, and Western Ontario and McMaster University Osteoarthritis Index (WOMAC) scores and range of motion (ROM) of the knee were measured at baseline, 2 weeks, 6 weeks, and 3 months.

**Results:**

The 100 mm VAS score for pain was significantly lower in the viscosupplementation group at 2 weeks post-surgery (27.5 mm vs. 40.7 mm, *P* = 0.047). ROM was significantly greater in the viscosupplementation group than in the control group at 2 weeks (131.5**°** vs. 121.0**°**, *P* = 0.044) post-surgery. No significant differences were observed in the IKDC or in the Tegner, Lysholm, and WOMAC scores between the two groups.

**Conclusions:**

Viscosupplementation after arthroscopic partial meniscectomy significantly reduced pain at 2 weeks post-surgery and improved ROM of the knee at 2 weeks post-surgery. There might be some benefits in terms of pain and functional recovery of viscosupplementation after arthroscopic surgery.

**Study design:**

Randomized controlled trial; Level of evidence, 1.

**Trial registration:**

This randomized controlled trial was registered at cris.nih.go.kr #KCT0004921.

## Introduction

Knee arthroscopy is one of the most commonly performed procedures in South Korea and the United States [1, 2]. Among knee arthroscopy, partial meniscectomy is one of the most performed surgeries. During arthroscopic surgery, continuous irrigation is performed for the visualization of the knee joint. However, irrigating fluids, such as saline, are known to negatively influence articular cartilage, [3] as they can alter the ideal environment of the knee joint by changing the temperature, osmolarity, and pH of the synovial fluid [4]. Their use during arthroscopic surgery may cause an increased removal of proteoglycans from the knee joint, exposing articular cartilage to possible mechanical damage [5]. This damage combined with surgical trauma may negatively affect management during the postoperative period following arthroscopic surgery. Collagens (mainly type II collagen), proteoglycans (mostly aggrecan), and other non-collagenous proteins (such as link protein, fibronectin, and cartilage oligomeric matrix protein) and smaller proteoglycans are all found in articular cartilage. The tissue’s compressive and tensile strength, which resists applied force in vivo, is due to the interaction between highly negatively charged cartilage proteoglycans and type II collagen fibrils [6].

Synovial fluid is a biological lubricant as well as a biochemical pool through which nutrients and regulatory cytokines pass through natural joints. Proteoglycan 4, hyaluronan, and surface-active phospholipids are molecules thought to play a crucial role in lubricating, either alone or in combination [6, 7]. The natural replacement of synovial fluid after arthroscopic surgery can be delayed by at least several days, which is the turnover time of synovial fluid. Administering hyaluronic acid (HA) or viscosupplementation to the joint immediately after surgery has been shown to affect the altered joint environment, resulting in reduced postoperative pain and accelerated functional improvements such as range of motion (ROM) of the knee, weight bearing and movement [8–11]. This concept seems to be probable considering the rheological properties of HA. Hyaluronan solutions are viscoelastic, and viscosity is strongly affected by shearing. Hyaluronan’s rheological characteristics have been linked to joint lubrication [6]. However, there are conflicting results regarding the effect of viscosupplementation after arthroscopic surgery. While some studies have reported positive effects in postoperative pain relief and expediting functional improvement, [10, 11] others have denied any benefit in using viscosupplementation after arthroscopic surgery [12, 13].

This study aimed to analyze the efficacy of single-dose intra-articular HA injection on pain and functional recovery after arthroscopic partial meniscectomy. We hypothesized that using intra-articular HA after arthroscopic partial meniscectomy would result in less pain and superior functional recovery.

## Methods

### Study design

In a single center, patients who had been scheduled to undergo arthroscopic partial meniscectomy were randomized. This single-blind, prospective, randomized controlled trial compared the clinical outcomes of a viscosupplementation versus control group between March 2020 and March 2021. This trial was registered at cris.nih.go.kr #KCT0004921. The study protocol was approved by the Institutional Review Board of Kyung Hee University Hospital. (KHUH 2020–03-041).

### Study population

Eligibility was assessed for patients aged ≥19 years who had been scheduled for arthroscopic partial meniscectomy. Indication for meniscectomy are meniscus tears that are unrepairable with symptoms that are resistant to conservative treatment, symptoms that interfere with daily activities, mechanical symptoms [14]. The exclusion criteria for this study were as follows: (1) osteoarthritis detected on preoperative imaging (Kellgren-Lawrence grade 2 or more), (2) focal cartilage lesion with International Cartilage Repair Society grade 3 or 4 detected during knee arthroscopic surgery, and (3) concurrent articular lesion requiring surgery (ligament, cartilage, or bony surgery). Patient groups were homogeneous for age, sex, BMI, preoperative range of motion of the knee. In the viscosupplementation group 16 patients underwent medial partial meniscectomy and 7 patients underwent lateral partial meniscectomy. In the control group 14 patients underwent medial partial meniscectomy and 10 patients underwent lateral partial meniscectomy.

### Study interventions

Patients were randomly assigned to two groups, the viscosupplementation group and the control group. For both groups, arthroscopic partial meniscectomy was carried out by a single experienced orthopedic surgeon, using a 5.5-mm, 30° arthroscope and a pressure-controlled irrigation system. Damaged or degenerative portion that was mobile was removed via motorized instruments and manual instruments. “Partial” was defined as resection of the meniscus to less than half of the total length with the peripheral rim intact [15]. In the viscosupplementation group, after drying the joint using suction via a shaver, HA (Jointseal 5 mg/10 mL; Lumenbio) was injected through the portal, under direct arthroscopic vision, to guarantee intra-articular delivery. Next, the skin portals were sutured, and no drainage was placed. In the control group, the joint was dried, the skin portals were sutured, and no drainage was performed.

Patients were allowed to ambulate with full weight-bearing on the day of the surgery. Gradual active and passive ROM and quadriceps strengthening exercises were implemented.

### Randomization

The patients were randomized using a random number generator with permuted blocks of 10. Allocations were kept in sequentially numbered and sealed in envelopes, which were opened the day before the surgery, after the patient provided written consent. The research coordinator and the patients were blinded to the treatment allocation.

### Study outcome

Knee pain during weight-bearing was measured on 100 mm visual analogue scale (VAS) for pain assessment at 1 day before the surgery and at 1 day, 2 weeks, 6 weeks, and 3 months post-surgery. The International Knee Documentation Committee (IKDC) subjective, Tegner, Lysholm, and Western Ontario and McMaster University Osteoarthritis Index (WOMAC) scores and the passive ROM of the knee were measured at 1 day before the surgery, 2 weeks, 6 weeks, and 3 months. The IKDC subjective score is a validated and self-administered questionnaire designed for patients with a variety of knee disorders that assesses knee function, symptoms, and ability to engage in sports activities ROM of the knee was defined as the maximal degree of flexion of the knee.

### Sample size and statistical analysis

The sample size was estimated to detect a 1-point difference in the postoperative pain VAS, which was considered clinically meaningful, using an independent *t*-test. Based on the data from a previous study, at least 25 patients in each group were required to detect this difference. The type 1 error and power were 0.05 and 0.8, respectively [16]

Descriptive data are reported as mean ± standard deviation for continuous values. Continuous variables between the two groups that were continuous, normally distributed, and homoscedastic were compared using the Student’s *t*-test. The Mann-Whitney test was used for data of ROM. and differences in other categorical variables between the two groups were analyzed using the chi-square test and the Fisher’s exact test. Differences during the postoperative follow-up period in both groups were analyzed using one-way analysis of variance. Statistical significance was set at *P* < 0.05. All statistical analyses were performed using SPSS (version 22.0; SPSS Corp., Chicago, USA).

## Results

**Fig. 1:**
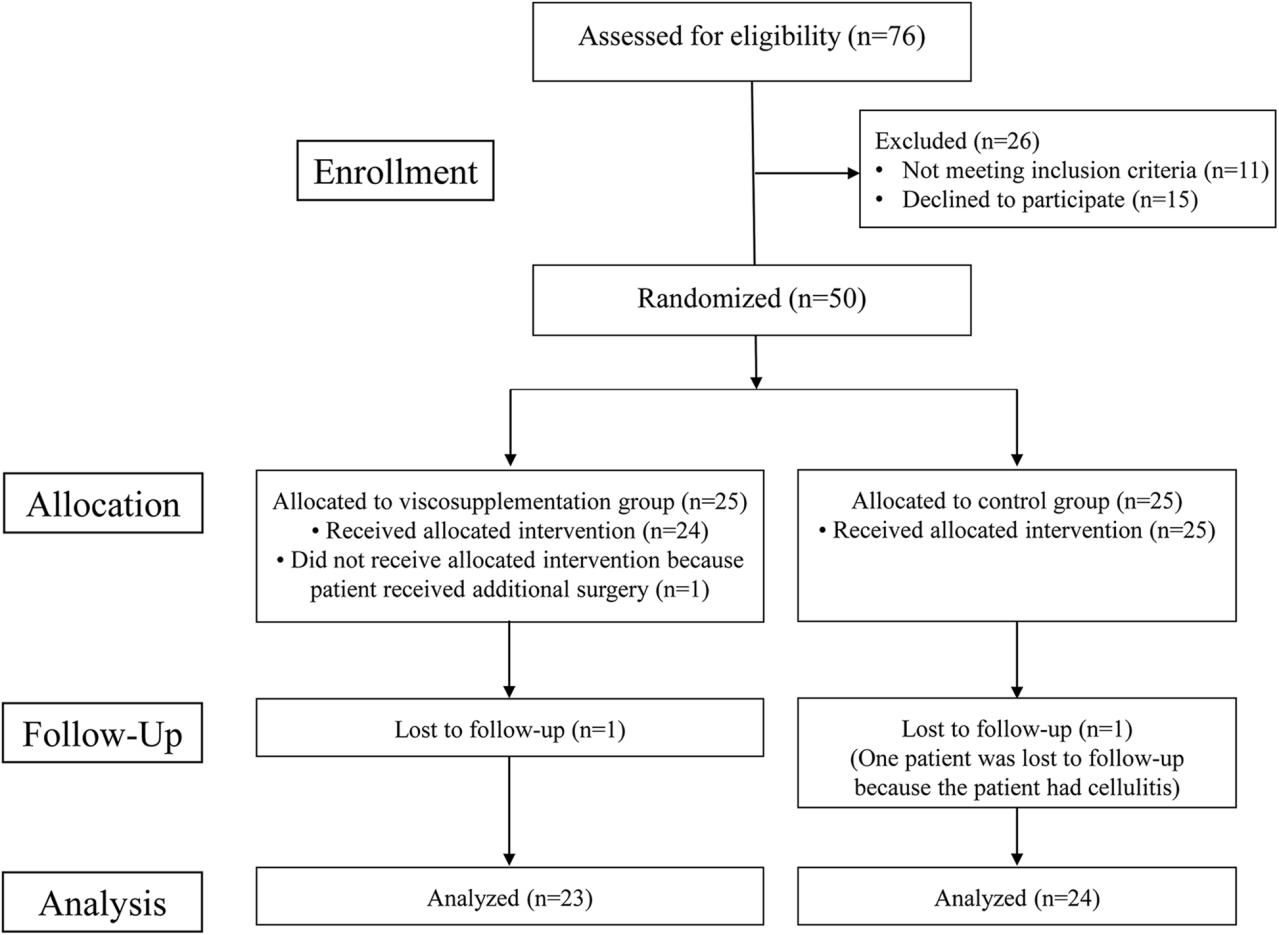
Flow chart of the study population

Out of a total of 76 patients that were screened for eligibility, 50 patients met the inclusion criteria and were randomized between March 2020 and January 2021. One patient in the viscosupplementation group did not receive the allocated intervention because the patient underwent additional surgery. Another patient in the viscosupplementation group was lost to follow-up. One patient in the control group had a surgical site infection and was excluded from the study (Fig. 1). There were no significant demographic differences between the two groups (Table 1).

**Table 1:** Baseline characteristics of patients

	Viscosupplementation group (*n* = 23)	Control group (*n* = 24)	*P*-value
Male/female	15 / 8	17 / 7	n.s
Age (year)	47.7 ± 13.2	46.7 ± 14.3	n.s
BMI (kg/m^2^)	27.0 ± 3.6	26.2 ± 4.9	n.s
ROM (°)	139.3 ± 2.3	137.1 ± 7.5	n.s
Medial/Lateral meniscus	16 / 7	14 / 10	n.s
Right/Left	12 / 11	18 / 6	n.s

The data are expressed as the mean ± standard deviation

*BMI* Body mass index, *ROM* Range of motion, *n.s* Not significant

### 100 mm VAS for pain

**Fig. 2:**
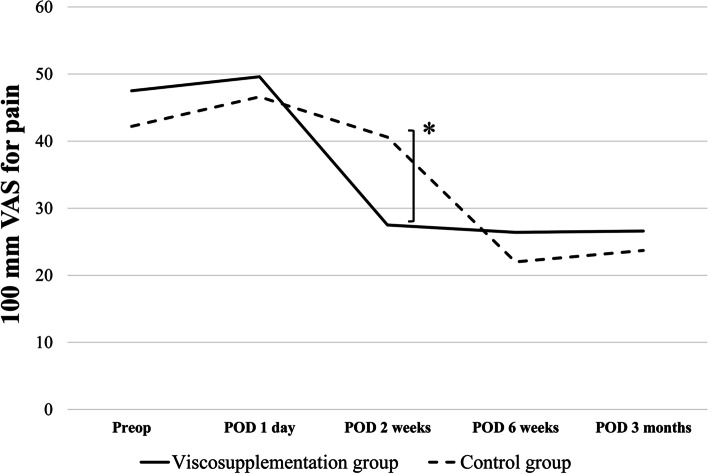
This figure represents the results of the 100 mm visual analog scale between the two groups at each time. The 100 mm visual analog scale for pain was significantly lower (Viscosupplementation group 27.5 mm ± 17.9 mm vs. Control group 40.7 mm ± 24.7 mm, *P* = 0.047) Student’s t-test was used to compare the data Asterisks indicate values that were significantly different between the two groups (*P* < 0.05).

Patients in the viscosupplementation group experienced significantly lower pain, as determined by the 100 mm VAS for pain at 2 weeks post-surgery, compared to those in the control group (27.5 mm ± 17.9 mm vs. 40.7 mm ± 24.7 mm; *P* = 0.047) (Fig. 2). There were no differences in the 100 mm VAS scores for pain between the two groups at 1 day, 6 weeks, and 3 months post-surgery.

### IKDC subjective, Tegner, Lysholm, and WOMAC scores

Both the preoperative and postoperative scores for IKDC, Tegner, Lysholm, and WOMAC were not significantly different between the two groups (Table 2).

**Table 2:** Comparison of clinical outcomes between the viscosupplementation group and the control group

	Preoperative	2 weeks Post-surgery	6 weeks Post-surgery	3 months Post-surgery
IKDC subjective score				
Viscosupplementation group	37.4 ± 11.9	43.6 ± 13.8	54.5 ± 12.4	54.4 ± 15.9
Control group	41.9 ± 15.0	45.4 ± 16.2	50 ± 15.0	53.2 ± 17.2
*P*-value	n.s	n.s	n.s	n.s
Lysholm score				
Viscosupplementation group	48.3 ± 17.2	57.1 ± 20.8	67.9 ± 17.8	66.8 ± 22.7
Control group	48.2 ± 21.4	59.0 ± 21.3	62.5 ± 23.3	65.6 ± 23.8
*P*-value	n.s	n.s	n.s	n.s
Tegner score				
Viscosupplementation group	3.7 ± 1.8	3.7 ± 1.6	4.5 ± 2.2	4.2 ± 1.8
Control group	4.4 ± 2.2	3.4 ± 1.8	4.1 ± 2.1	4.8 ± 2.0
*P*-value	n.s	n.s	n.s	n.s
WOMAC score				
Viscosupplementation group	27.5 ± 14.9	23.4 ± 15.7	14.6 ± 12.0	16.7 ± 14.9
Control group	23.9 ± 12.9	24.4 ± 15.4	21.5 ± 20.5	15.6 ± 14.3
*P*-value	n.s	n.s	n.s	n.s

The data are expressed as the mean ± standard deviation

*IKDC* International Knee Documentation Committee, *WOMAC* Western Ontario and McMaster University Osteoarthritis Index, *ROM* Range of motion, *n.s* Not significant

### Range of motion

**Fig. 3:**
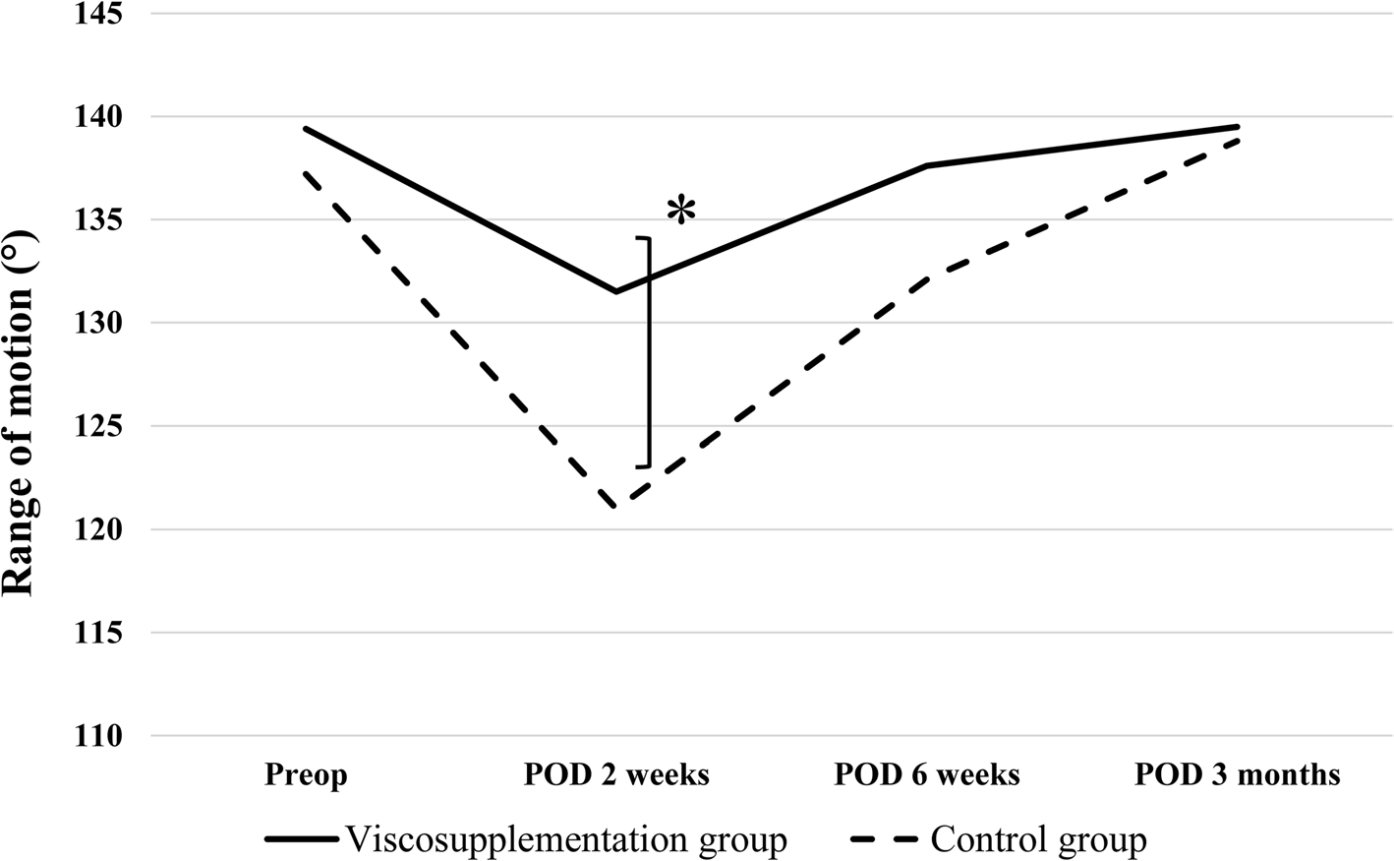
This figure represents the results of the range of motion between the two groups at each time. Range of motion was significantly greater in the viscosupplementation group at 2 weeks post-surgery compared to the control group. (Viscosupplementation group 131.5° vs Control group 121.0°, *P* = 0.044) Mann-Whitney test was used to compare the data. Asterisks indicate values that were significantly different between the two groups (*P* < 0.05)

Patients in the viscosupplementation group showed greater ROM than those in the control group at 2 weeks post-surgery (Fig. 3). At 2 weeks post-surgery, the ROM of the viscosupplementation group was significantly greater than that of the control group (131.5° ± 9.7° vs. 121.0° ± 22.8°; *P* = 0.044).

## Discussion

The most important finding of the present study is that postoperative use of viscosupplementation after arthroscopic partial meniscectomy significantly reduced pain 2 weeks after surgery and resulted in better ROM at 2 and 6 weeks after surgery.

The justification for viscosupplementation is based on the biological and mechanical properties of HA, which function as a lubricant and mechanical shock absorber, increasing the rheological properties of synovial fluid. HA is also known to decrease proinflammatory activity [13]. Based on these characteristics, HA could be beneficial in a variety of therapeutic situations where the synergistic effects could give rapid symptomatic relief and functional recovery.

Previous clinical trials have shown conflicting results regarding the efficacy of intra-articular HA injection with some trials showing superior results. Chen et al. reported that the knee muscle strength index and VAS pain score were significantly better when using sodium hyaluronate after arthroscopy of the knee [17]. Mathies et al. also reported that using sodium hyaluronate immediately after arthroscopy reduced postoperative pain and swelling [16]. Huang et al. reported that hyaluronan injection after anterior cruciate ligament reconstruction resulted in more functional muscle rehabilitation [18]. Lastly, Anand et al. reported that using sodium hyaluronate after arthroscopy showed better pain scores and favorable WOMAC scores at 3 and 6 weeks post-surgery [11]. In contrast, a randomized controlled trial by Filardo et al. reported that early viscosupplementation after arthroscopic partial meniscectomy failed to provide any benefit in terms of functional recovery and pain control [13]. Many studies show conflicting results. One explanation maybe because amount of HA used for the viscosupplementation differ between studies. The present study and the study by Anand et al. used single injection of HA (5 mg / 10 mL), study by Filardo et al. used a single injection of HA (24 mg /3 mL).

The present study showed that viscosupplementation resulted in better pain scores at 2 weeks post-surgery, but showed no difference at 6 weeks and 3 months post-surgery. Previous studies have shown that the half-life of hyaluronan molecules in cartilage is 2–3 weeks. Our hypothesis is that the viscosupplementation performed immediately after surgery may act for only 2–3 weeks, resulting in decreased pain, especially 2 weeks after surgery [19]. The difference between the two groups may diminish after the natural synovial fluid plays its role.

Early functional rehabilitation and a nearly unrestricted start of weight-bearing and full range of motion are the current trends of rehabilitation after partial meniscectomy [20, 21]. After partial meniscectomy, pain is a major limiting factor for patients before returning to full activity [14, 22]. In light of the current trend of early functional rehabilitation, this study provides some benefit to patients. Although viscosupplementation did not provide a difference in pain and functional recovery at 3 months post-surgery, there was a difference in pain at 2 weeks post-surgery. As pain is a major limiting factor for patients in rehabilitation, reducing pain at postoperative week 2 is not an insignificant achievement. The present study demonstrated that the ROM was better at 2 weeks after surgery in the viscosupplementation group than in the control group.

We believe that viscosupplementation is not a “troubleshooter” that can solve every problem after arthroscopic partial meniscectomy. There are many reasons that may contribute to a patient having poor outcomes after surgery that viscosupplementation cannot solve, such as whether the patient is obese or has undergone a large amount of meniscal resection. However, we speculate that after conducting this clinical trial, it may have some beneficial effects in terms of pain and functional recovery during the early postoperative period (the first 2 weeks), which in turn may lead to earlier rehabilitation.

### Limitations

This study has some limitations. First, the surgeon was not blinded to the study. Second, even though bias was eliminated, the amount of resected meniscus was difficult to quantify. As the extent of meniscal resection is related to worsened clinical outcomes, this factor may have affected the results of this clinical trial. In addition, medial meniscectomy tends to result in better outcomes than lateral meniscectomy. Future studies focusing on medial or lateral meniscectomy should be performed [23]. Third, this study included a small sample. A future study with a larger number of samples is warranted for a more definitive conclusion.

## Conclusions

In conclusion, this prospective randomized clinical trial showed that viscosupplementation after arthroscopic partial meniscectomy significantly reduced pain at 2 weeks post-surgery and improved ROM at 2 weeks post-surgery. Therefore, viscosupplementation may provide some benefits in terms of pain and functional recovery after arthroscopic surgery.

## Data Availability

Data and materials can be accessed through a request to the lead author.
